# Bidirectional Modulation of Substantia Nigra Activity by Motivational State

**DOI:** 10.1371/journal.pone.0071598

**Published:** 2013-08-06

**Authors:** Mark A. Rossi, David Fan, Joseph W. Barter, Henry H. Yin

**Affiliations:** 1 Department of Psychology and Neuroscience, Duke University, Durham, North Carolina, United States of America; 2 Department of Neurobiology, Duke University, Durham, North Carolina, United States of America; 3 Center for Cognitive Neuroscience, Duke University, Durham, North Carolina, United States of America; University of Chicago, United States of America

## Abstract

A major output nucleus of the basal ganglia is the substantia nigra pars reticulata, which sends GABAergic projections to brainstem and thalamic nuclei. The GABAergic (GABA) neurons are reciprocally connected with nearby dopaminergic neurons, which project mainly to the basal ganglia, a set of subcortical nuclei critical for goal-directed behaviors. Here we examined the impact of motivational states on the activity of GABA neurons in the substantia nigra pars reticulata and the neighboring dopaminergic (DA) neurons in the pars compacta. Both types of neurons show short-latency bursts to a cue predicting a food reward. As mice became sated by repeated consumption of food pellets, one class of neurons reduced cue-elicited firing, whereas another class of neurons progressively increased firing. Extinction or pre-feeding just before the test session dramatically reduced the phasic responses and their motivational modulation. These results suggest that signals related to the current motivational state bidirectionally modulate behavior and the magnitude of phasic response of both DA and GABA neurons in the substantia nigra.

## Introduction

Motivational state is a key determinant of behavior. Food-seeking behavior, for example, persists until the animal is sated. A variety of satiety signals have been studied in the brain, especially in the hypothalamus [1]. There is also an extensive literature on the neural basis of reward seeking behavior [2], implicating the basal ganglia and ascending dopaminergic projections from the midbrain. But it is not clear whether, and how, satiety signals from homeostatic feeding control circuits can influence basal ganglia circuits mediating goal-directed behavior [2].

One possibility is that satiety signals can directly alter the activity of midbrain dopamine (DA) neurons [3], [4]. As a major neuromodulator, DA regulates synaptic transmission and plasticity in the basal ganglia [5], [6]. It has been implicated in action selection [7], [8], attention [9], learning [10], [11], [12], [13], and motivation [14], [15], [16], [17], [18]. Phasic activity of DA neurons was reported to follow the presentation of a food reward or a stimulus that predicts the reward [19], [20]. Specific depletion of DA in the dorsal striatum, a target of the nigrostriatal DA pathway, can also disrupt the motivational control of feeding behavior [4].

DA neurons of the SN pars compacta (SNC) are highly connected with the SN pars reticulata (SNR), a major output nucleus of the basal ganglia [21]. SNR outputs can inhibit DA neurons, while the release of DA can also alter the firing patterns of SNR GABA neurons [5]. The SNR contains GABAergic neurons that project to motor initiation regions in the brainstem and the thalamus. It appears to play a major role in selecting the appropriate behaviors based on the current motivational state of the animal [22], [23]. But the functional relationship between DA and GABA neurons in the substantia nigra is poorly understood.

To study the effects of motivational state on the SNR GABAergic output of the basal ganglia and the SNC dopaminergic input to the basal ganglia, we recorded from both regions simultaneously using multi-electrode arrays as hungry mice became sated on a fixed time reward schedule. In this task, a cued food reward is delivered once a minute, resulting in progressive and controlled satiation of the animal and reduced reward seeking behavior. We hypothesized that shifts in motivational states can also alter the output of the basal ganglia by dynamically shaping the activity of the substantia nigra neurons. We found populations of DA and GABA neurons whose activity was bidirectionally modulated by the motivational state of the animal.

## Materials and Methods

### Ethics Statement

All procedures were approved by the Institutional Animal Care and Use Committee at Duke University and followed National Institutes of Health guidelines (Protocol Number: A062-11-03).

### Subjects

Male C57BL/6J mice (Jackson Laboratories) aged 2–4 months (*n* = 7) were used. During testing, food was restricted to maintain the mice at approximately 85% of free-feeding body weight. Water was available at all times in the home cages. All experiments were conducted during the dark phase of the animal's light cycle, in accordance with the Duke University Institutional Animal Care and Use Committee guidelines.

### Surgery

Mice were anesthetized with isoflurane (induction at 3%, maintained at 1%) and head fixed on a stereotax (Kopf). A craniotomy (∼1 mm by 2 mm) above the site targeting the right SN was created. Dura was removed, and the microarray was slowly lowered into place, targeting the final coordinates (in mm relative to Bregma): AP −3.2, ML+1.2, DV −4.6. Microarrays (Innovative Neurophysiology) were 2×8 arrays of 7 mm-long, 50 µm-diameter tungsten recording electrodes (5 mm reference electrode) with 150 µm electrode spacing and 200 µm row spacing attached to an Omnetics connector. Microarrays were grounded to screws placed in the skull using a 0.008″ silver ground wire and then fixed in place with dental acrylic. Mice were allowed to recover for at least one week following surgery before the start of recording.

### Histology

**Figure 1:**
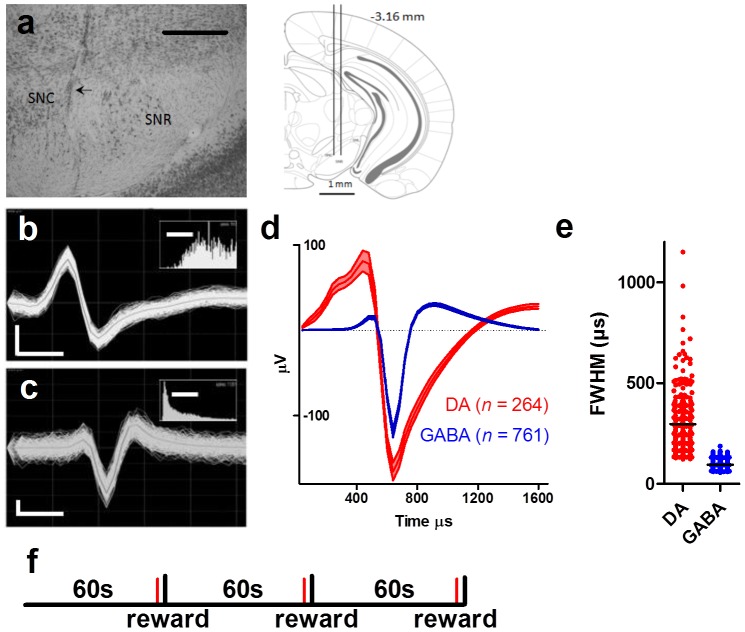
Single unit recording in substantia nigra. (**a**) Representative photomicrograph of electrode tracks in the pars compacta (SNC; arrow; left). Schematic representation of the electrode placement (right; SNR, pars reticulata). Coordinates are relative to Bregma. Sample traces of DA (**b**) and GABA (**c**) neurons showing the narrower spike waveform of GABA neurons. Insets are inter-spike interval histograms. (**d**) Average waveforms (±s.e.m.) for all recorded DA and GABA neurons. We classified the neurons based on the waveforms of their action potentials. DA neurons, for example, are characterized by longer spike durations than GABA neurons. *n* is indicated by the numbers. (**e**) Full width half max (FWHM) values of classified DA and GABA neurons. (**f**) Schematic illustration of the FT60 task. One pellet was delivered into the food cup every 60 s (vertical black lines). An auditory cue preceded the pellet delivery by ∼550 ms (red lines). Vertical scale bars represent 68 µV (**b**) and 23 µV (**c**), and horizontal scale bars represent 500 µm in **a**, and 200 µs in **b** and **c** (10 ms in insets).

Following the completion of all behavioral experiments, mice were deeply anesthetized with isoflurane and transcardially perfused with 0.9% saline followed by 10% buffered formalin solution. Brains were sliced into 80 µm coronal sections with a Vibratome 1000 Plus, stained with thionin, and examined with a light microscope to verify placement of the electrode tips within SN [24] (**Fig. 1a**).

### Behavior

Behavioral testing took place in a Med Associates operant chamber (35 cm×28 cm×22 cm) designed for *in vivo* electrophysiology and housed inside light-resistant and sound-attenuating walls as described previously [25], [26]. The walls and floor grid were made of Plexiglas with a food cup in the center of one wall, into which 14 mg food pellets (Bio-Serv rodent purified diet) were dispensed. On the wall opposite the food cup was a 3 W, 24 V house light. Before testing each day, a 16-channel head stage (Blackrock Microsystems) was connected to the array, and a single cable extended from the head stage to a motorized commutator (Dragonfly R&D Inc) that allowed the mouse to rotate freely. At the start of fixed time 60 s (FT60; *n* = 7 mice; mean 13.9±3.15 s.e.m. sessions per mouse) testing, the house light was illuminated, and one pellet was delivered into the food cup every 60 s for two hours. The pellet dispenser made a distinct and audible sound ∼550 ms before the pellet was available to the mouse, serving as a cue predicting reward. After 120 pellets had been delivered, the house light was turned off, and the session ended. During testing, entries into the food cup were recorded using an infrared photo beam located just inside the entrance to the food cup. Each time the beam was broken, one entry was recorded using computers with the Med-PC-IV program (Med Associates). During extinction testing (2 mice, 4 experiments), the procedure was the same except the pellets were not dispensed into the food cup, but the dispenser still sounded. During pre-feeding sessions (4 mice, 9 experiments), mice were given free access to food pellets for a minimum of 1 hour or until they consumed at least 0.75 g.

### Data Collection and Analysis

Single unit activity was recorded using the Cerebus data acquisition system (Blackrock Microsystems) [27]. Neurons were selected based on activity observed prior to the start of testing on each day. Data were filtered with analog and digital band-pass filters (analog high-pass 1^st^ order Butterworth filter at 0.3 Hz, analog low-pass 3^rd^ order Butterworth filter at 7.5 kHz). Single unit data were separated with a high-pass digital filter (4^th^ order Butterworth filter at 250 Hz) and sampled at 30 kHz. Single units were processed using online sorting algorithms and then re-sorted offline (Offline Sorter, Plexon). Only single-unit activity with clear separation from background noise was used for analyses [28], [29]. Single units were analyzed using NeuroExplorer (Nex Technologies) and custom software (Matlab).

### Cell type classification

**Figure 2:**
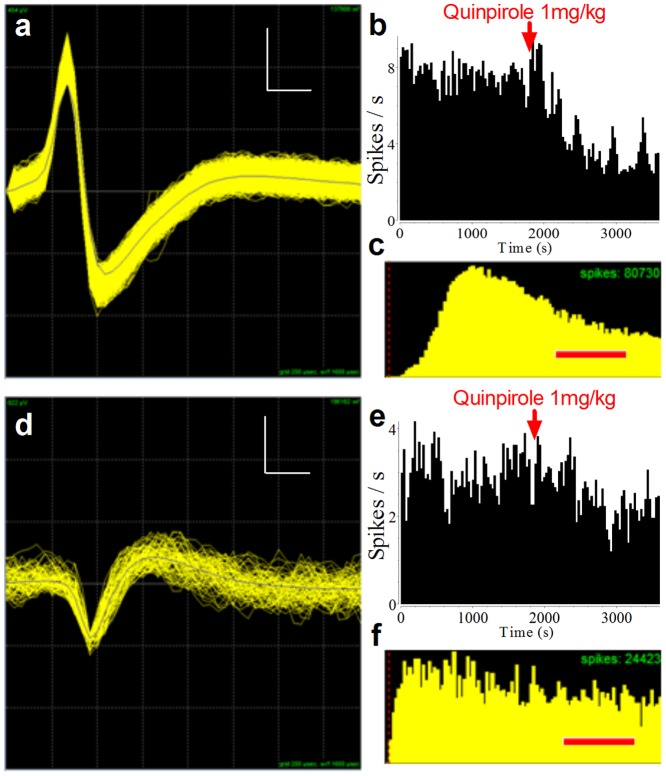
Effects of quinpirole on dopaminergic and GABAergic neurons. To confirm the classification of cell types, we performed additional pharmacological experiments. Quinpirole (1 mg/kg), a D2 receptor agonist, was injected intraperitoneally during a recording session (red arrows). Example responses of DA (**a**–**c**) and GABA (**d**–**f**) neurons to quinpirole treatment are shown. Immediately following injection, the rate of dopaminergic firing decreased (**b**), whereas GABAergic neurons were unaffected (**e**). (**c, f**) Inter-spike interval histograms for the neurons in **a** and **d**. In **a** and **d**, vertical scale bars represent 76 µV and 137 µV, respectively. Horizontal scale bars represent 200 µs. Scale bars in **c** and **f** represent 25 ms.

Neurons were classified as GABAergic or dopaminergic based on spike duration. Spike duration was measured using the full width half-max value, or the width of the valley at half of the maximum depth (**Figs. 1d**–**e**) [22]. These sorting parameters were then tested using injections of the dopamine D2 receptor agonist, quinpirole. Quinpirole (1 mg/kg; Sigma) was injected i.p. (4 experiments in 3 mice) while neural activity was being recorded. Neurons that were classified as dopaminergic based on the duration of their waveforms were confirmed to be dopaminergic when the rate of firing was reduced by quinpirole. Neurons classified as GABAergic did not reduce their firing rate following quinpirole injection (**Fig. 2**).

### Population JPSTH analysis

Only neurons recorded simultaneously and on different electrodes were included in the analyses to avoid shadowing effects [30]. To obtain population joint peri-stimulus time histograms (pJPSTH), we first calculated the raw JPSTH by plotting the peri-reward spikes of each pair of neurons (raw JPSTH; one on the vertical axis and one on the horizontal axis). The predictor was defined as the cross product of the PSTHs of each pair of neurons. Both the raw JPSTHs and predictors were calculated with sliding bins of 100 ms. The predictor matrix was then subtracted from the raw JPSTH of each pair of neurons bin by bin to control for correlations between spike trains due to co-variation in spike rates, generating the ‘corrected’ JPSTH [31], [32]_ENREF_26. Each bin of the corrected JPSTH was then normalized by the standard deviation of the trial to trial response (SD of predictor matrix) [31]. The pJPSTH was obtained by averaging the normalized JPSTHs. Diagonal mean and s.e.m. values were obtained from the corrected but non-normalized pJPSTHs using a width of 500 ms.

### Video Tracking

Videos were recorded at 30 frames per second and analyzed offline. Using custom software (Matlab), the mouse's horizontal and vertical position within each frame were tracked. The position data were then smoothed using a moving average filter (boxcar average of 30 ms). Mouse speed was calculated as the derivative of the position data. This was low-pass filtered at 50 pixels/frame, and missing points were interpolated to remove any artifacts.

### Statistical analysis

All recorded neurons were tested for significant modulation following the onset of the reward cue. Paired *t*-tests compared the firing rate during the 500 ms just preceding the reward cue to the 500 ms immediately following reward cue (phasic window) on each trial. Neurons whose firing rate following the reward cue was significantly greater than the firing rate during the 500 ms preceding the cue (*p*<0.05) were classified as showing a phasic response. Modulation was determined using the number of spikes in the phasic window from each trial pooled into 10-trial blocks. A one-way ANOVA was performed on the twelve 10-trial blocks using the number of spikes in each trial as independent samples. Neurons were classified as modulated if there was a significant effect of block (*p*<0.05). Significantly modulated neurons were then tested to assess directionality of the change by comparing the average values of the first and last blocks. ‘Increasing’ neurons were those modulated neurons in which the average response from the first block of 10 trials was lower than the response of the last block. ‘Decreasing’ neurons showed the opposite pattern.

## Results

### Classification of DA and GABA neurons

Using 16-electrode arrays, we recorded from DA (*n* = 264) and GABA (*n* = 761) neurons in the SN from 7 male mice. GABA neurons can be distinguished from DA neurons on account of their narrower spike waveforms and higher tonic rate of firing (**Fig. 1**, DA 4.2±0.29 Hz, GABA 8.1±0.33 Hz, mean ±s.e.m.). The classification of DA and GABA neurons was based on visual inspection of their waveforms, especially the spike duration. To confirm classification, we also used a pharmacological manipulation. We injected mice with quinpirole, which activates D2-like DA autoreceptors on the presynaptic terminals of DA neurons and presumably reduces the firing rate of DA but not GABA neurons [33], [34] (**Fig. 2**, 4 tests in 3 mice; 18 GABA, 13 DA neurons). In all tested cases, the firing rates of neurons that were classified as DA were reduced by the quinpirole injection (*t*_(12)_ = 4.98, *p*<0.001), whereas neurons classified as GABA showed no significant reduction (GABA: *t*_(17)_ = 1.68, *p*>0.05).

### Behavior

Food deprived mice (maintained at ∼85% of free feeding body weight) were trained under a fixed time schedule of reward. Under this schedule, a 14 mg food pellet (Bio Serv, rodent purified diet) was delivered into a food cup every 60 s (FT60) for two hours (**Fig. 1f**). The distinct sound of the pellet dispenser predicted the presence of the pellet in the cup. The mice learned to enter the food cup as soon as the sound was detected. The session length and inter-reward interval were determined by previous work, which showed that the mice could finish all the pellets (∼1.68 g total) in the allotted time. After each session, we confirmed that each mouse was able to finish the pellets.

### Phasic activity and synchrony following reward cue

**Figure 3:**
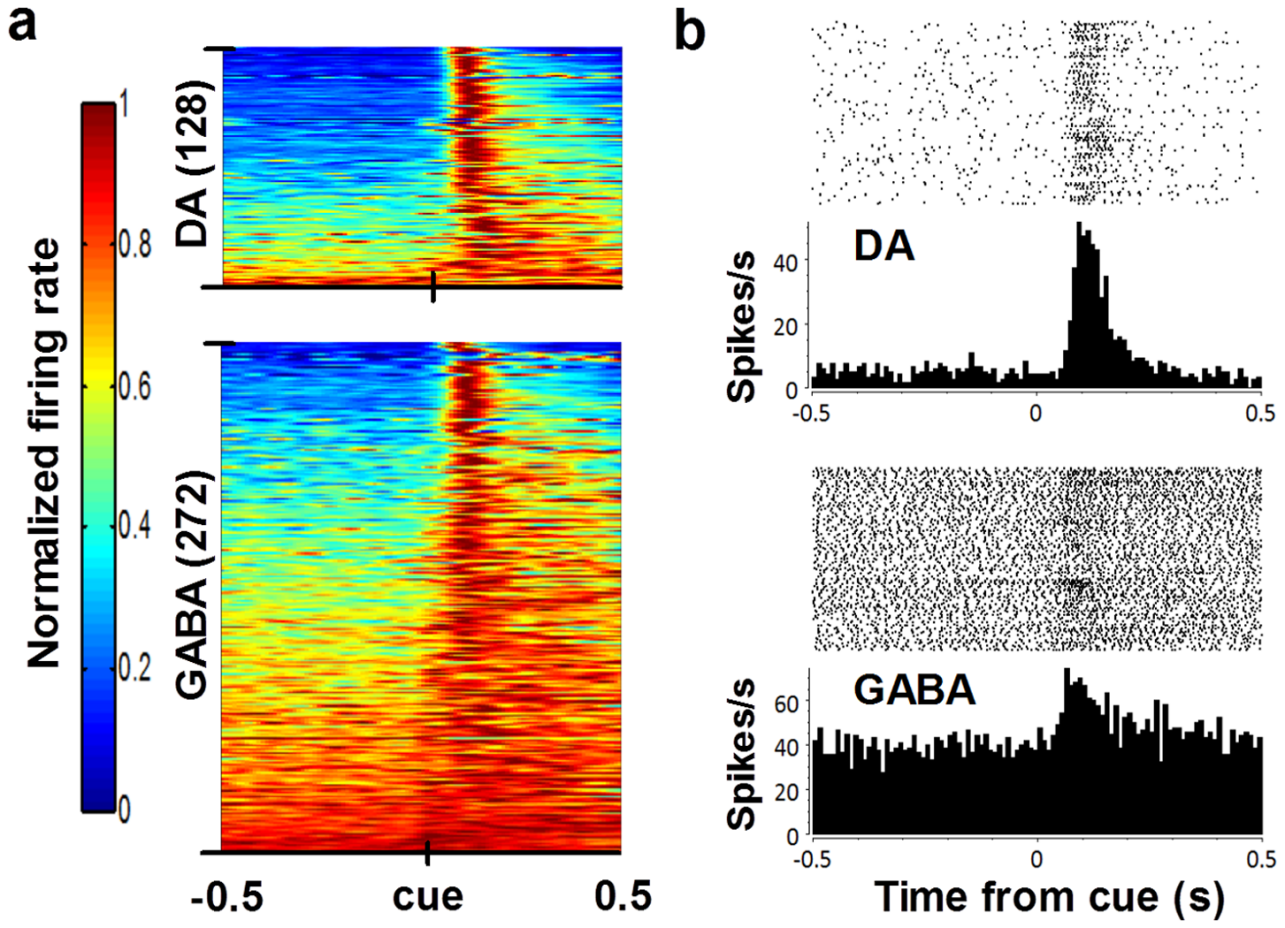
Dopaminergic and GABAergic neurons showed a phasic response to reward cue. (**a**) Activity of neurons that increased following reward cue normalized to the maximum firing rate; sorted according to increasing cumulative maximum values. Each row represents one neuron. X axis is time from reward cue onset (s). *n* is indicated in parentheses. (**b**) Representative peri-event histograms and rasters for individual DA (top) and GABA (bottom) neurons showing phasic responses following the reward cue (10 ms bins).

The delivery of the pellet elicited a phasic response in many neurons we recorded (**Fig. 3**). Approximately 63% (128/202) of DA neurons and 45% (272/605) of GABA neurons recorded during FT60 sessions showed a significant increase in firing rate immediately following the reward cue (paired *t-*test, *p*<0.05). Some neurons showed significant inhibition immediately following the cue (10 DA, 35 GABA; *p*<0.05). Only those neurons showing significant excitation following the reward cue were included in further analyses.

**Figure 4:**
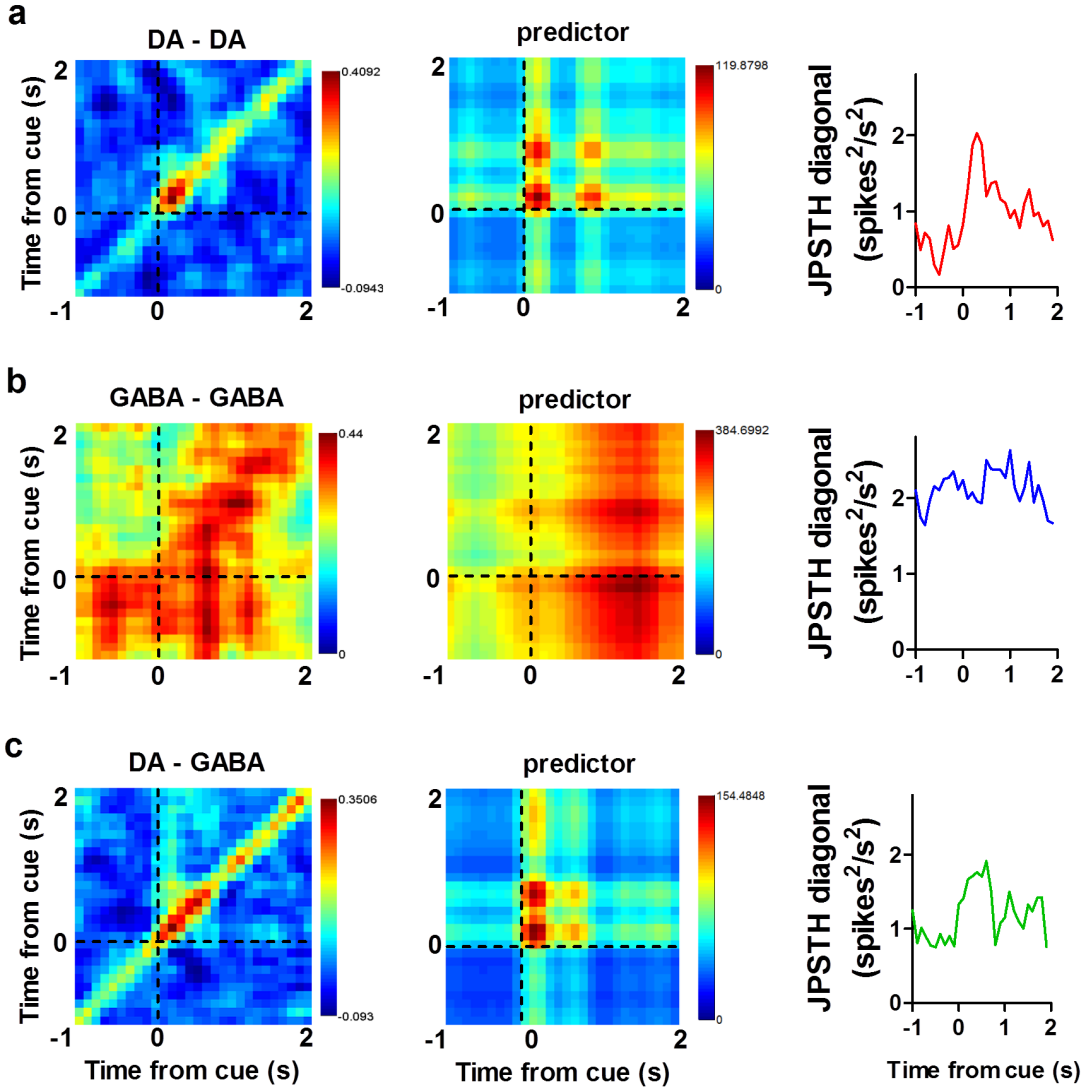
Synchrony is enhanced immediately after the reward cue. Examples of synchrony between pairs of SN neurons following the reward cue. (**a**) Example of a corrected joint peri-stimulus time histograms (JPSTH) in the left column, corresponding predictor matrix (middle) and histogram of JPSTH diagonal (right; 500 ms width) for a pair of simultaneously recorded DA neurons (**a**), GABA neurons (**b**), and DA-GABA pairs (**c**); DA activity is plotted on the horizontal axis. Axes are time relative to the reward cue. The predictor matrix is subtracted bin-by-bin from the raw JPSTH, and then normalized by the standard deviation of the predictor matrix (100 ms×100 ms bins).

**Figure 5:**
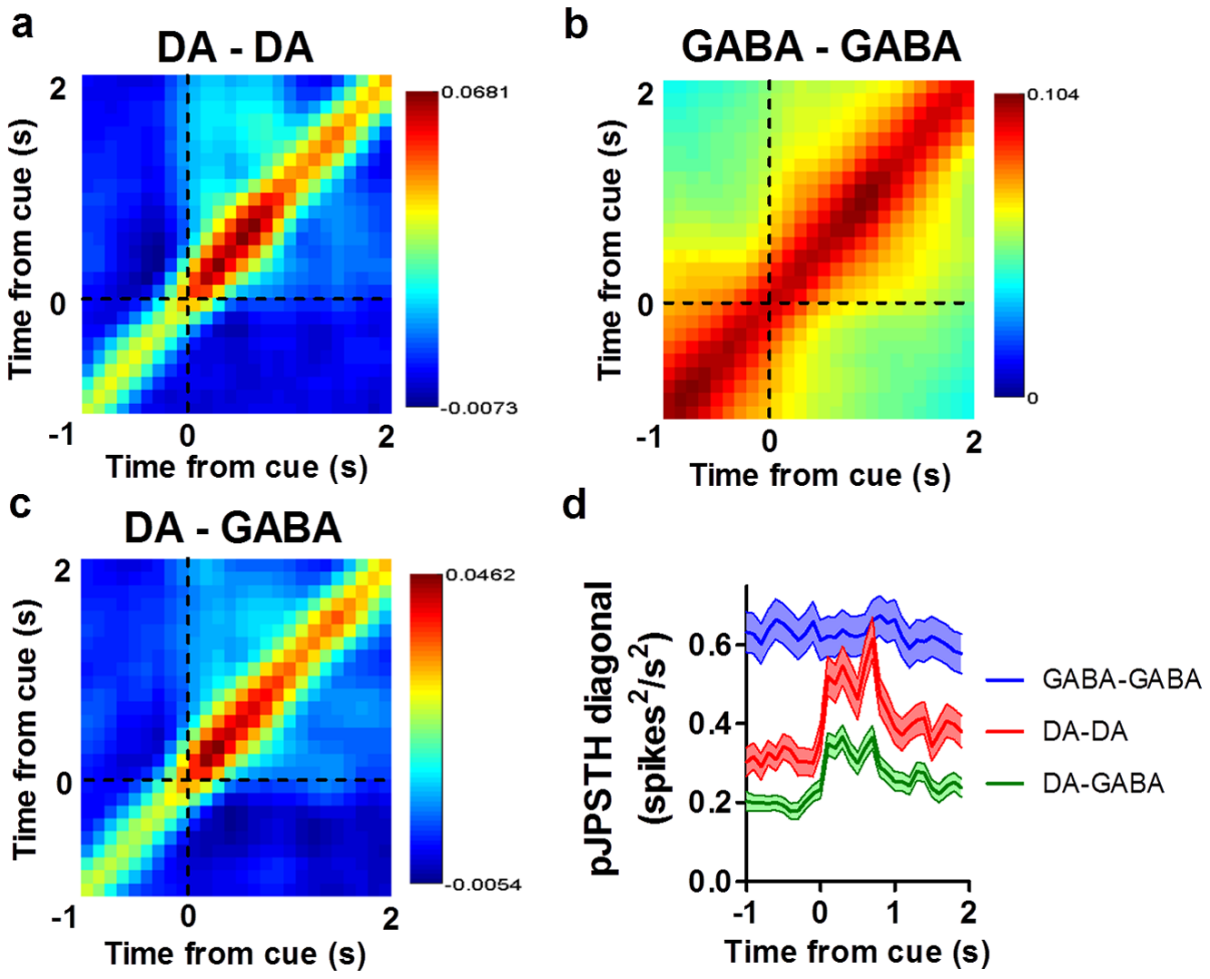
Population JPSTH analysis. (**a**) Normalized population JPSTH (pJPSTH) of all DA-DA pairs (*n* = 185 pairs). Synchrony was enhanced immediately after the onset of the cue. (**b**) Synchrony among GABA-GABA pairs (*n* = 475 pairs) was not affected by the reward cue. (**c**) Synchrony between DA and GABA neurons (*n* = 394 pairs) was enhanced following the reward cue. pJPSTH bins are 100 ms×100 ms. Values are normalized by the standard deviation of predictor matrices. (**d**) The diagonal of each corrected pJPSTH is plotted (width  = 500 ms, bins  = 100 ms). DA-DA and DA-GABA pairs showed enhanced synchrony following the cue, whereas cue-elicited synchrony was not observed among GABA-GABA pairs. Only those neurons showing significant excitation following the reward cue were included in the analysis.

We also found synchrony in simultaneously recorded nigral neurons following the reward cue (**Figs. 4**, **5**). We used joint peri-stimulus time histograms (JPSTH) to assess how synchrony between pairs of neurons changes following the cue [31]. We first calculated individual JPSTH for simultaneously recorded pairs of neurons that showed a phasic burst following the reward cue (**Fig. 4**) [30]. Each predictor matrix (**Fig. 4** **middle column**) was subtracted from the raw JPSTHs to account for synchrony that may occur due to co-variation in spike trains. This yielded an estimate of cue-induced synchrony for pairs of neurons (**Fig. 4** **right column**). Individual JPSTHs were then pooled to produce a population JPSTH (pJPSTH; **Fig. 5**). We found that simultaneously recorded pairs of DA neurons showed enhanced synchrony following the reward cue (**Figs. 4a**, **5a**). In contrast, although pairs of GABA neurons showed strong synchrony, this synchrony was not affected by the reward cue (**Figs. 4b**, **5b**). Synchrony between DA and GABA neurons was also enhanced after the cue (**Figs. 4c**, **5c**–**d**; two-way ANOVA, Cell type x Time, Interaction: *F*_(58,31530)_ = 0.92, *p* = 0.65; Cell type: *F*_(2,31530)_ = 456.6, *p*<0.0001; Time: *F*_(29,31530)_ = 3.14, *p*<0.0001). Taken together, these results demonstrate a coordinated GABAergic and dopaminergic phasic response to a reward cue.

### Motivational modulation of neural activity

**Figure 6:**
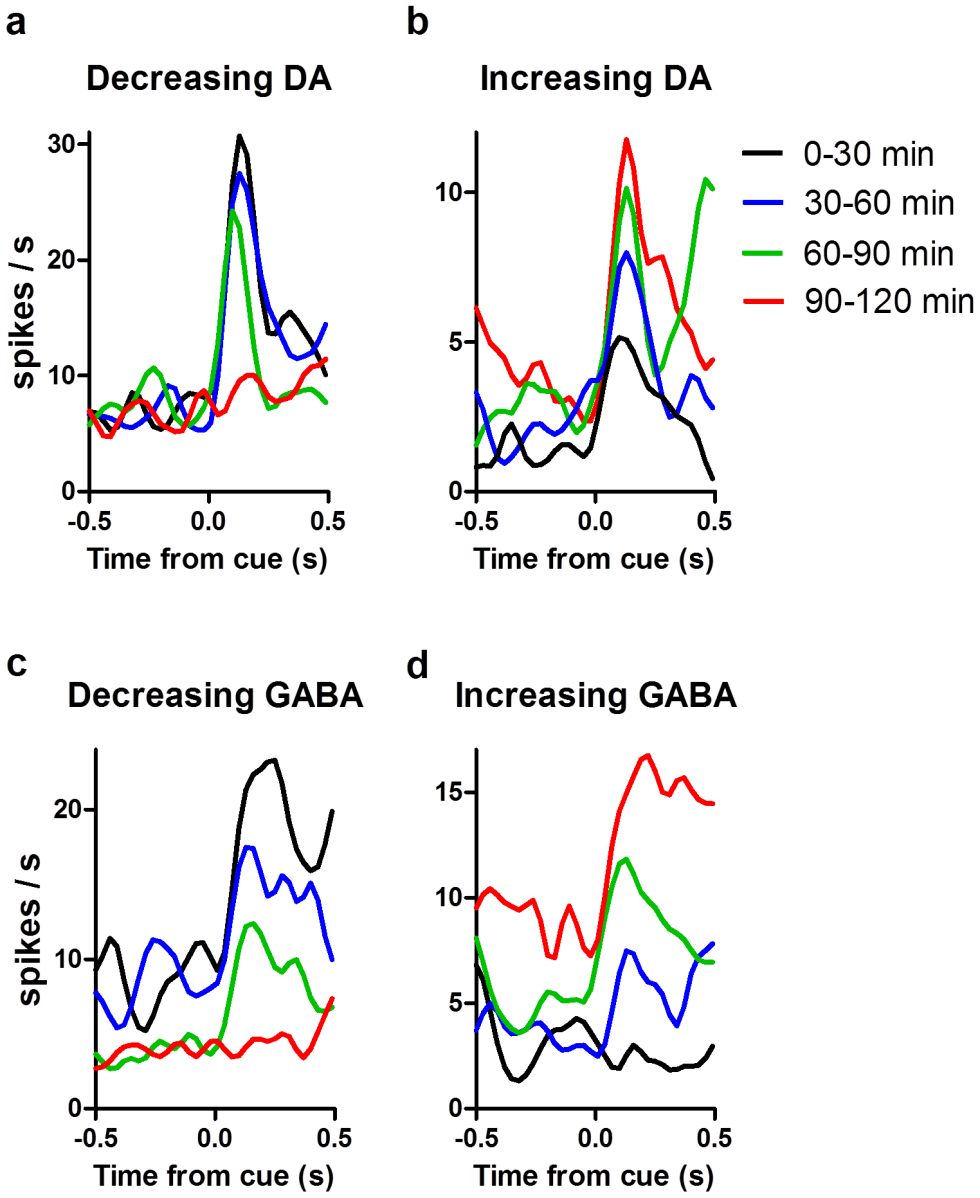
Motivational modulation of phasic responses to reward cue. Motivational modulation of DA and GABA activity in SN. (**a**) Sample PSTH illustrating the motivational shift in phasic cue response of decreasing (**a**) and increasing (**b**) DA neurons separated into four 30 min blocks (bin size  = 30 ms). GABA neurons also showed similar decreasing (**c**) and increasing (**d**) motivational modulation.

To assess the role of this coordinated neuronal response to the reward cue and its relationship to food-seeking behavior, we examined changes in neural activity as the animals became sated during the session. In both DA and GABA neuron populations, we identified two distinct types of motivational modulation: neurons that increased phasic activity as the animals became sated (‘increasing’; n = 68 GABA, n = 19 DA) and others that decreased phasic activity as animals became sated (‘decreasing’; *n* = 138 GABA, *n* = 74 DA) (**Fig. 6**).

### Stability of single unit recording during each session

**Figure 7:**
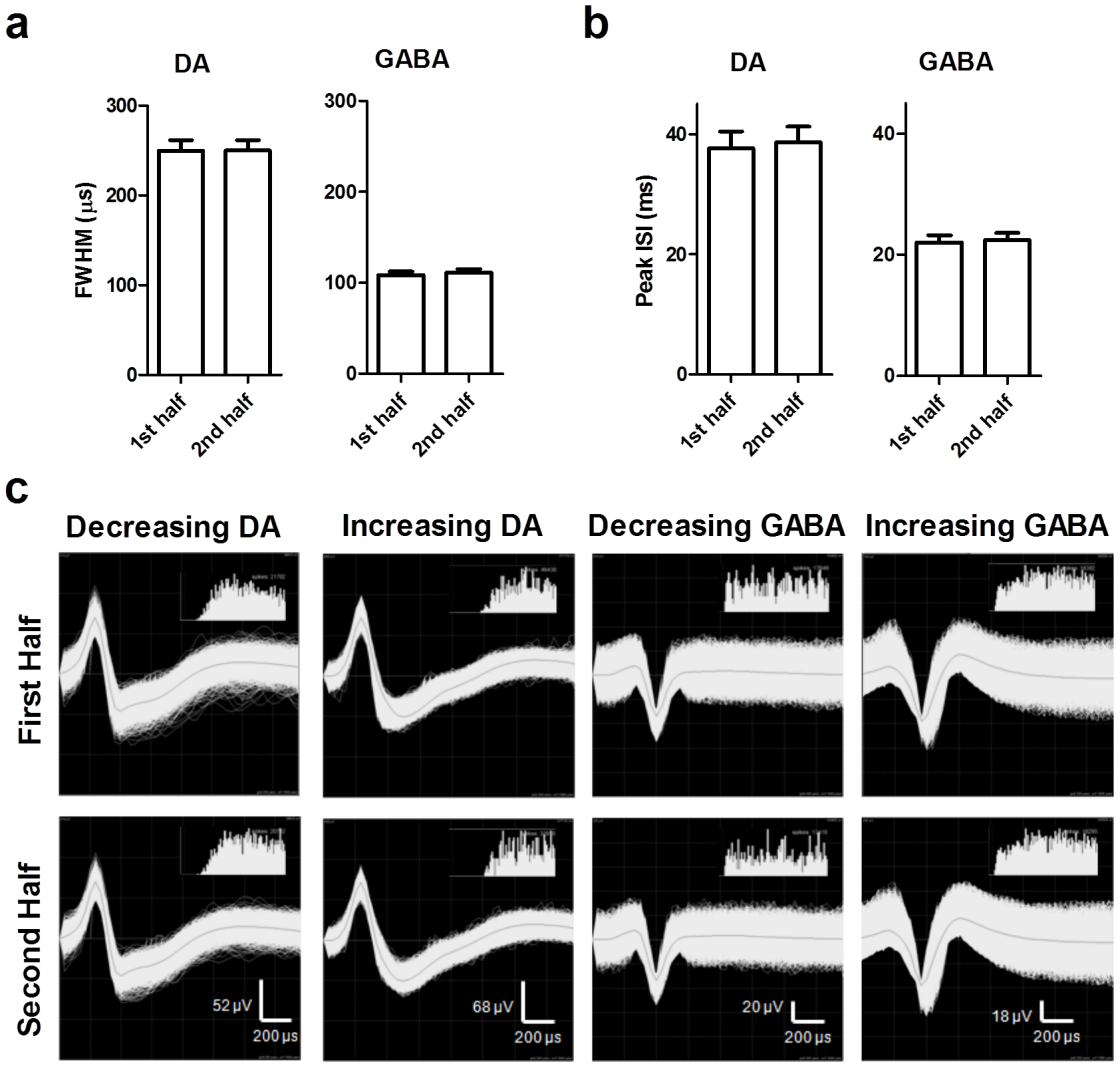
Modulation is not due to changes in neuron isolation. To test whether changes in neuronal isolation could account for the modulation observed in single unit activity, waveform characteristics were calculated for all modulated neurons for each half of the recording session. Neither FWHM (**a**) nor the peak of the inter-spike interval (ISI; **b**) changed during the two-hour recording sessions (paired *t*-tests, *p*>0.05). (**c**) Sample traces from the first and second half of the session for modulated neurons. The range of ISI values shown is 0–30 ms.

The observed effects could be attributed to lack of stability in the recording. To explicitly rule out this possibility, we examined the waveform characteristics of each modulated neuron during the first and second hours of the recording session (**Fig. 7**). Among the modulated neurons, neither the FWHM (**Fig. 7a**; paired *t*-test; DA: *t*_(116)_ = 0.06, *p*>0.05; GABA: *t*_(255)_ = 1.76, *p*>0.05) nor the peak of the inter-spike interval histogram (**Fig. 7b**; DA: *t*_(116)_ = 0.48, *p*>0.05; GABA: *t*_(255)_ = 0.40, *p*>0.05) changed during the recording session. There was no evidence to suggest that changes in recording stability contributed to the modulation observed.

### The effects of satiety, pre-feeding, and extinction

**Figure 8:**
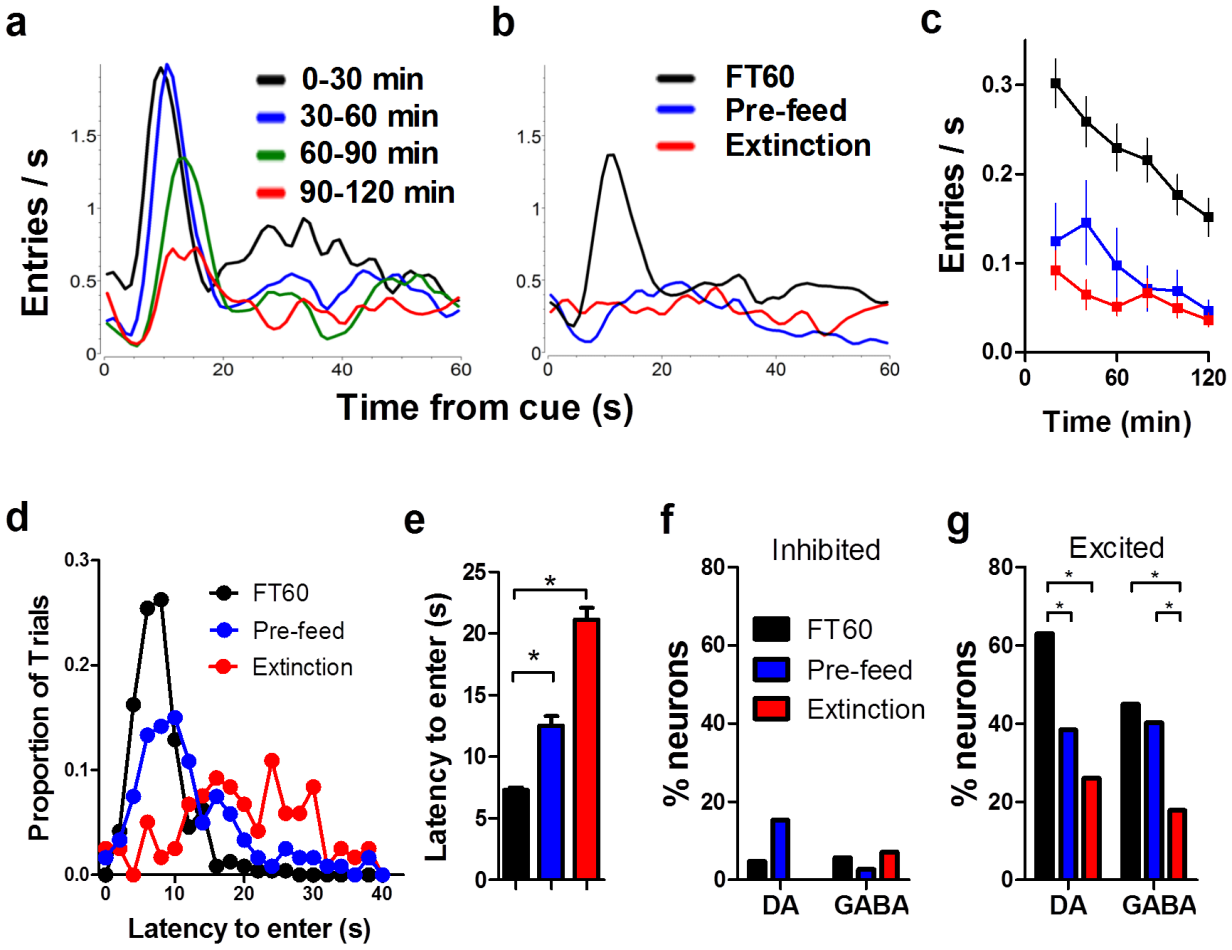
Food-seeking behavior gradually decreases with satiety. (**a**) Following reward delivery, mice entered the food cup to collect the pellet. Representative data from one mouse are shown. (**b**) The rapid increase in food cup entries after the reward cue was eliminated during extinction and following pre-feeding. Traces are from the same mouse. FT60 data is the average from all four blocks in **a**. (**c**) Rate of entries decreased as mice became sated during FT60 sessions. It was also reduced during extinction and following pre-feeding (average of all mice on all testing days; 20 min bins). (**d**) The latency to enter the food cup following reward delivery increased during extinction and following pre-feeding treatment. (**e**) Latency to enter the food cup for all mice on all testing days was pooled to yield population averages. (**f**) The proportion of DA and GABA neurons that exhibited significant inhibitions following the reward cue was unaffected by pre-feeding and extinction. (**g**) The number of excitatory responses varied as a function of motivational state for both DA and GABA neurons. * represents significant difference at alpha of 0.05.

**Figure 9:**
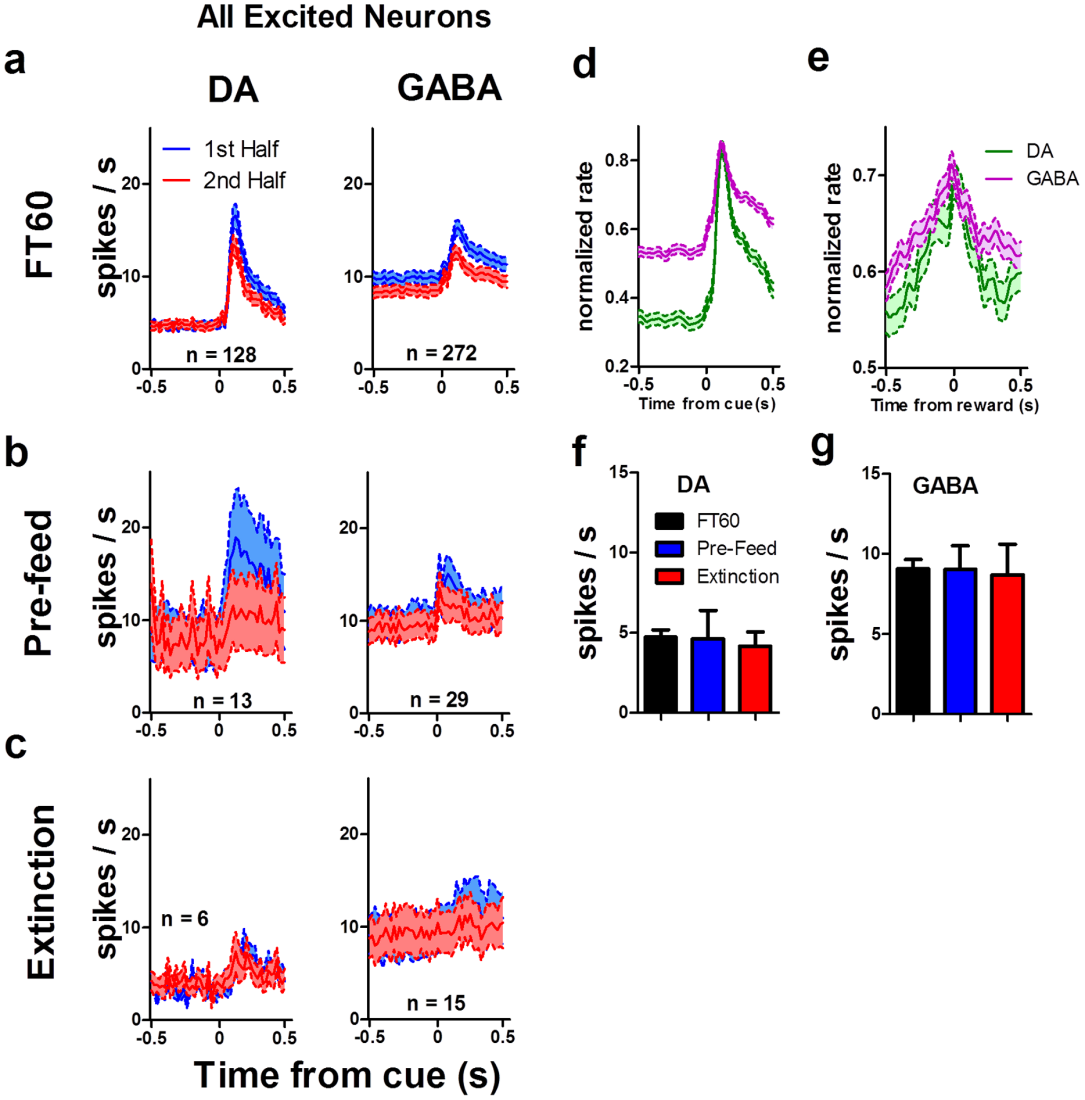
Average response of all excited neurons, including those that do not show motivational modulation during the session. To compare the basal activity and mean responses between the classes we examined the responses of all neurons that were excited following the cue. The activity (mean ±s.e.m.; 20 ms bins) of all neurons with excitatory responses to the reward cue, regardless of motivational modulation, is plotted for each half of FT60 (**a**), pre-feeding (**b**) and extinction (**c**) sessions. Overall, there was a reduction in the magnitude of the phasic response as mice became sated. During extinction (**c**) the phasic response is nearly abolished for both DA and GABA neurons. The response to the cue (**d**) and to reward receipt (**e**) are dissociable. The population responses are shown for DA (*n* = 128) and GABA (*n* = 272) neurons recorded during FT60 sessions. (**f**–**g**) Baseline firing rates do not vary between FT60, pre-feed, or extinction sessions for DA or GABA neurons presented in **a**–**c** (one-way ANOVAs, *F*<1.0, *p*>0.05).

During the session, all mice became sated and progressively reduced their food cup entries (**Figs. 8a,c**; One-way ANOVA, main effect of time on rate of entry; *F*_(5,72)_ = 29.25, *p*<0.0001). Overall the phasic response of both DA and GABA neurons was reduced as mice became sated (**Fig. 9a**).

If the increasing and decreasing neurons (**Fig. 6**) are indeed modulated by motivation, then we would not expect a similar pattern when the motivational state does not change significantly during the session. Pre-feeding the animals prevents significant changes in the motivational state by inducing satiety at the beginning of the recording session. Pre-feeding reduced the rate of food cup entries (**Figs. 8b,c**) and increased the latency to enter the food cup following reward delivery (**Figs. 8d,e**; one-way ANOVA, *F*_(2,354)_ = 91.22, *p*<0.0001). Pre-feeding also dramatically altered neural activity in the SN. The proportion of neurons that were inhibited (DA: 6/39; GABA: 2/72) following the reward cue was not affected (**Fig. 8f**; χ^2^ FT60 vs pre-feed; DA χ = 1.903, *p>*0.05, GABA χ = 1.47, *p*>0.05). The proportion of DA neurons excited by the reward cue was reduced by pre-feeding (**Fig. 8g**; (15/39); χ = 5.23, *p<*0.05), but the proportion of excited GABA neurons (29/72) was unaffected by pre-feeding (χ = 0.99, *p*>0.05).

While pre-feeding prevents changes in motivational state by inducing satiety in the mice immediately before the testing session, changes in motivational state can also be prevented using extinction. We recorded neural activity while the cue was still presented but no pellet was delivered into the food cup. The mice therefore remained hungry during the extinction session. Like pre-feeding, extinction had a dramatic effect on the behavior and neural activity. Extinction quickly reduced the rate of food cup entries (**Fig. 8c**) and increased the latency to enter the food cup following reward delivery (**Figs. 8d,e**). Extinction also reduced the magnitude of the phasic response (**Fig. 9c**) and the proportion of DA (6/23) and GABA (15/84) neurons that were excited following the reward cue (**Fig. 8g**; χ^2^ FT60 vs pre-feed; DA χ = 14.16, *p<*0.001, GABA χ = 8.70, *p*<0.01), without affecting the proportion of inhibited neurons (**Fig. 8f**; DA (0/23): χ = 2.51, *p>*0.05; GABA (6/84): χ = 0.21, *p*>0.05).

To determine whether changes in the magnitude of the phasic response of DA and GABA neurons reflected changes in motivational state, we compared the activity in the first half with activity in the second half of the recording session (**Fig. 9**). Because the phasic response was significantly reduced during pre-feeding and extinction sessions (**Figs. 8g** **,** **10**), we also included neurons that did not exhibit significant phasic excitation in the analyses to determine if there was motivational modulation of neuronal activity in the absence of a statistically significant phasic peak. During pre-feeding sessions, increasing DA neurons showed no modulation of activity, but decreasing neurons still showed evidence of modulation (**Fig. 11b**).

**Figure 10:**
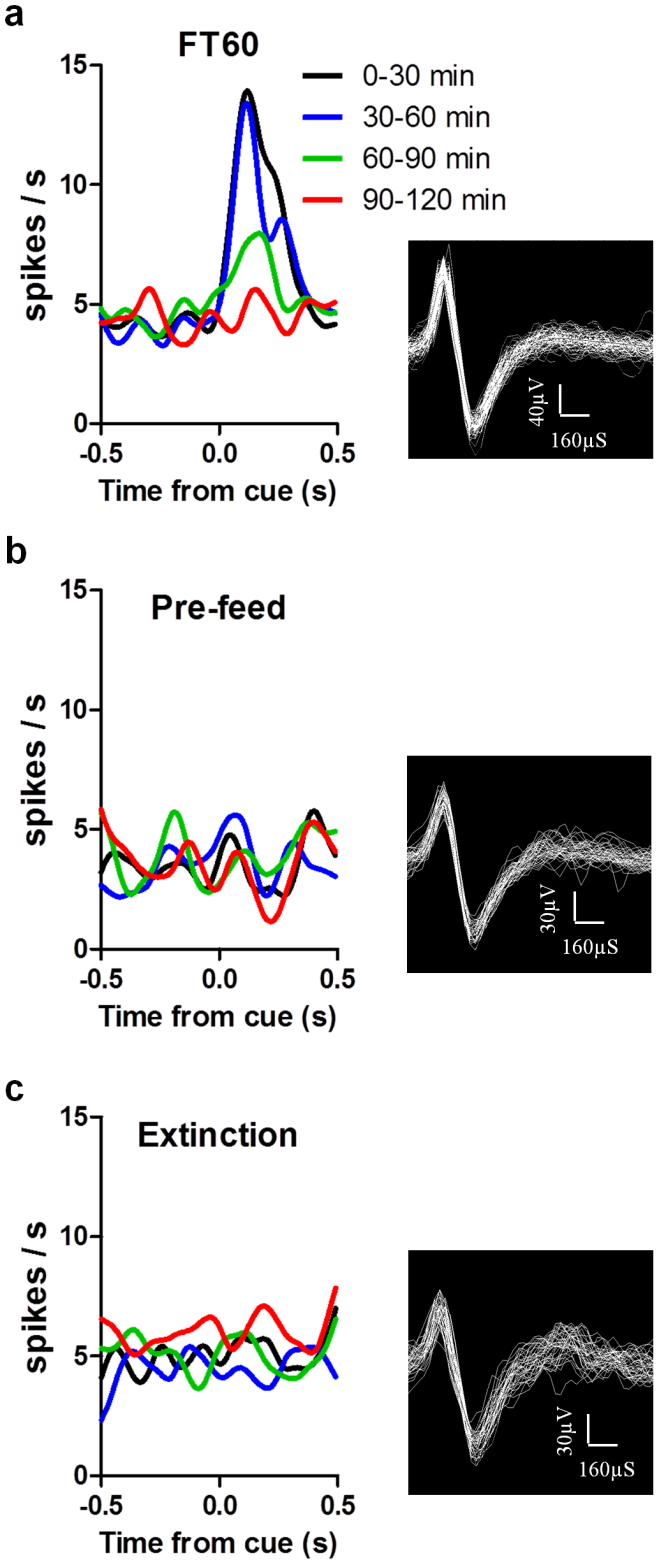
Motivational modulation is absent following pre-feeding and during extinction. of the response of a dopamine neuron to the cue in FT60 (**a**), pre-feed (**b**), and extinction (**c**) sessions. Each line represents the average response from a 30-trial block. Bins  = 10 ms, Gaussian smooth  = 100 ms. Corresponding waveforms are shown at the right.

**Figure 11:**
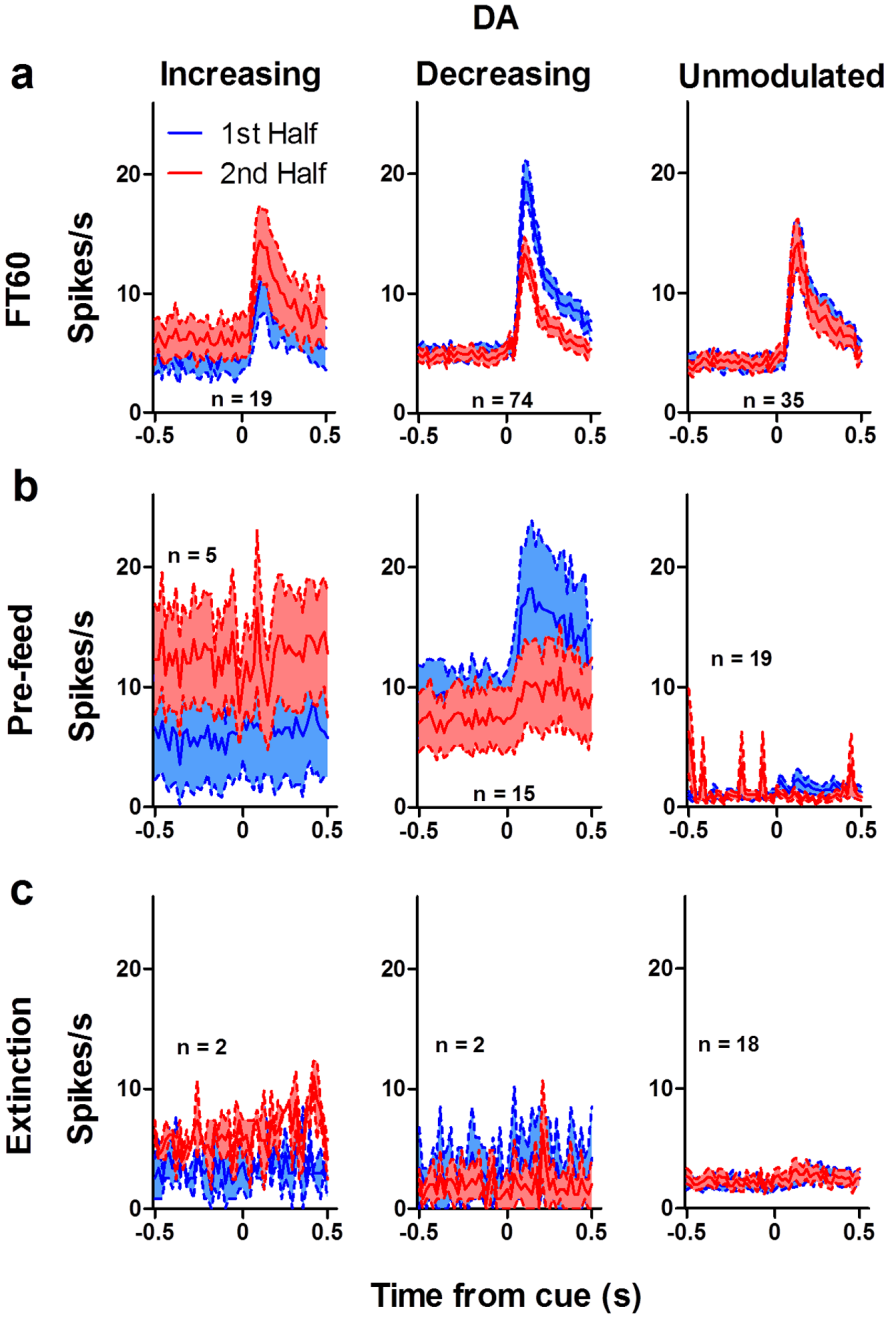
Summary of phasic response in dopamine neurons. To compare the magnitude of the satiety effect to the magnitude of the mean response, we plotted the responses of all dopamine neurons showing a cue-elicited phasic burst. (**a**) The phasic response to the cue of increasing DA neurons (*n* = 19) was higher for the second half of FT60 sessions than it was for the first half. Decreasing DA neurons (*n* = 74) showed the opposite pattern. The phasic response of unmodulated neurons (*n* = 35) did not change over time. Modulation of the phasic response was reduced by pre-feeding (**b**; *n* = 5 increasing, 15 decreasing, 19 unmodulated) and eliminated during extinction (**c**; *n* = 2 increasing, 2 decreasing, 18 unmodulated). The phasic response of increasing neurons was nearly abolished following pre-feeding and during extinction, and the phasic response of decreasing neurons was abolished during extinction.

**Figure 12:**
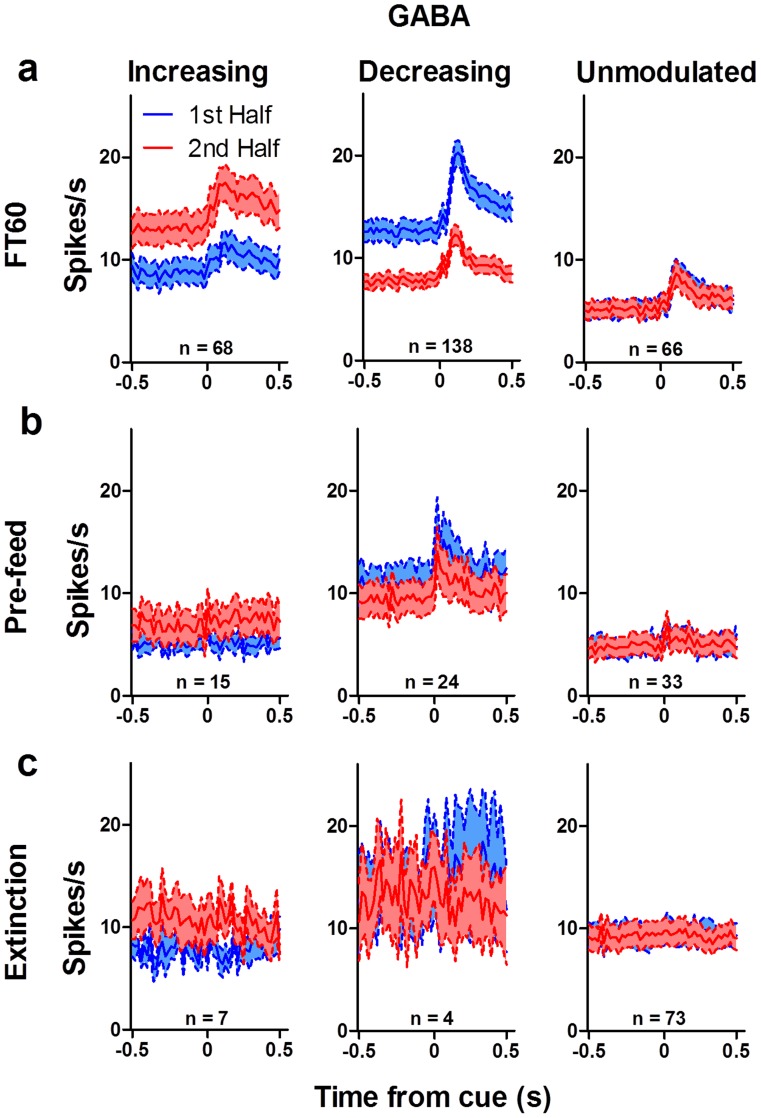
Summary of phasic response in GABA neurons. GABA neurons show the same pattern of modulation as DA neurons. (**a**) The phasic response to the reward cue of increasing GABA neurons (*n* = 68) was higher for the second half of FT60 sessions than it was for the first half. Decreasing GABA neurons (*n* = 138) showed the opposite pattern. The phasic response of unmodulated neurons (*n* = 66) did not change. Modulation of the phasic response was reduced by pre-feeding (**b**; *n* = 15 increasing, 24 decreasing, 33 unmodulated) and eliminated during extinction (**c**; *n* = 7 increasing, 4 decreasing, 73 unmodulated). The phasic response of increasing neurons was nearly abolished following pre-feeding and during extinction, and the phasic response of decreasing neurons was abolished during extinction.

**Figure 13:**
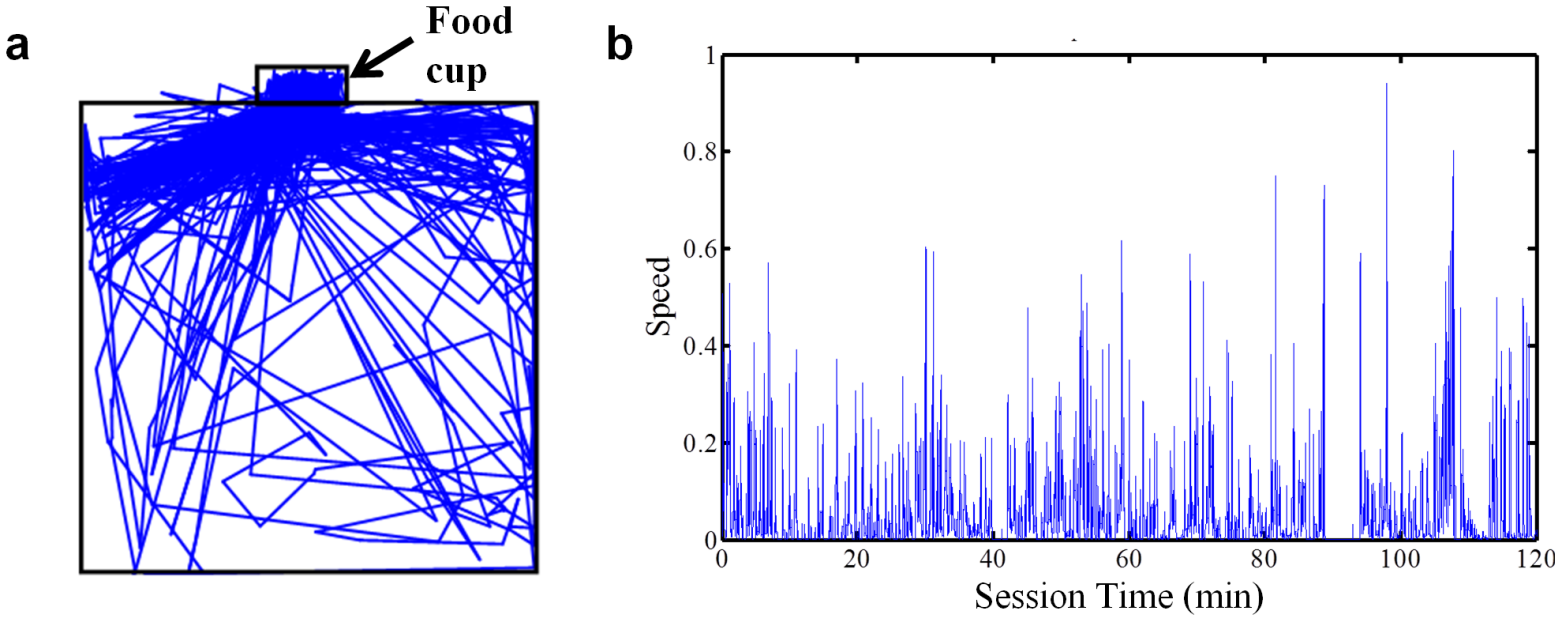
Mice are active throughout the recording session. To ensure that motivational modulation was not caused by mice falling asleep or losing interest in the pellets, we used video tracking to record the position of mice during recording sessions. Mice spent most of their time near the food cup (**a**). Speed was calculated (pixels per frame) throughout the session. Mice remained consistently active for the entire two-hour session (**b**).

Following pre-feeding, motivational modulation was much reduced (**Fig. 9**). During extinction (**Fig. 9c**), the phasic response in both DA and GABA neurons was almost completely abolished. Modulation of the neural activity was absent (**Figs. 11c**, **12c**). Thus, changes in the phasic response of both DA and GABA neurons may reflect changes in the motivational state of the animal. When animals are pre-fed to induce satiety before testing, modulation is reduced, but not entirely eliminated. This may be caused by the fact that mice are not completely sated at the start of the recording session. They typically consume some but not all of the pellets that are delivered, which may account for the presence of some modulation during pre-feeding sessions. Although the mice ate all the pellets during FT60 sessions, it is possible that the lack of overall activity towards the end of the session, rather than motivational state *per se*, may be responsible for the observed effects. That is, the mice were no longer active toward the end of the session, as they became sated. To examine this possibility, we used automated video tracking of mice to quantify their behavior during the recording session. Mice spent almost all of their time near the food cup (**Fig. 13a**), and their movement velocity did not change significantly during the session, though they entered the food cup less frequently with satiety (**Fig. 13b**).

### Motivational modulation of synchrony

**Figure 14:**
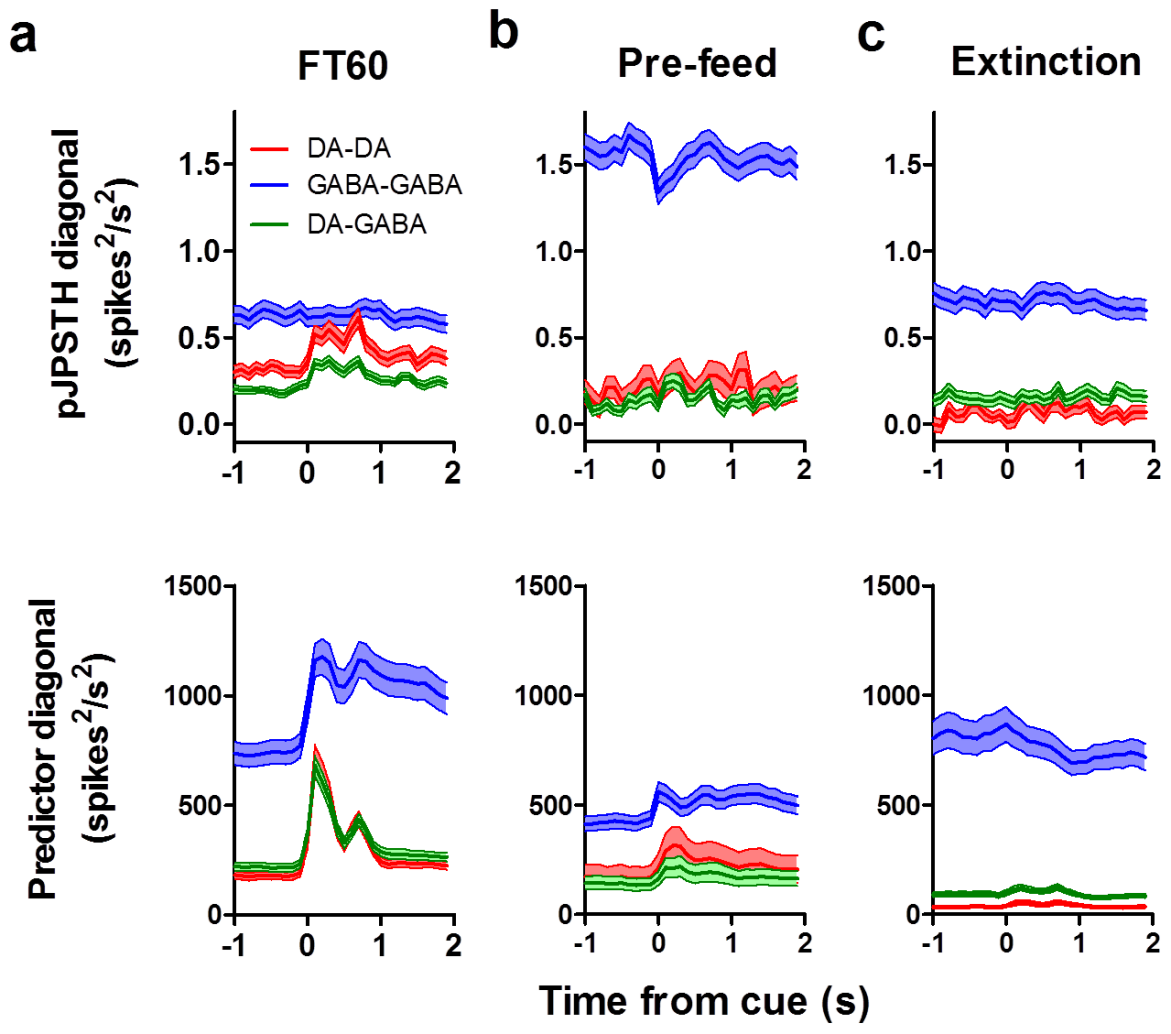
Cue-elicited synchrony of neural activity is also modulated by motivational state. pJPSTH diagonals (mean ±s.e.m.; 100 ms×100 ms bins; 500 ms diagonal width) are shown for DA-DA (*n = *185 FT60; *n* = 81 pre-feed; *n* = 45 extinction), GABA-GABA (*n = *475 FT60; *n* = 346 pre-feed; *n* = 389 extinction), and DA-GABA (*n = *394 FT60; *n* = 144 pre-feed; *n* = 138 extinction) pairs recorded during FT60 (**a**), pre-feeding (**b**), and extinction (**c**). The diagonals of the corrected and normalized pJPSTHs are in the top row, while the diagonals of the predictor matrices are in the bottom row. The predictor was defined as the cross-product of the JPSTH for each pair of neurons. Pre-feeding reduced cue-elicited synchrony for DA-DA and DA-GABA pairs while increasing overall synchrony between GABA-GABA pairs (**b**). Extinction reduced synchrony between DA-GABA and DA-DA pairs without affecting GABA-GABA pairs (**c**).

We next examined how cue-evoked synchrony varied as a function of motivational state. We found cue-evoked synchrony between DA-DA and DA-GABA pairs during FT60 sessions (**Fig. 14a**), but both pre-feeding (**Fig. 14b**; two-way ANOVA [Cell Type x Time], Interaction: *F*_(58,17040)_ = 0.36, *p* = 1.00; Cell Type: *F*_(2,17040)_ = 1908, *p*<0.0001; Time: *F*_(29,17040)_ = 0.36, *p* = 1.00) and extinction (**Fig. 14c**; two-way ANOVA [Cell Type x Time], Interaction: *F*_(58,17070)_ = 0.09, *p* = 1.00; Cell Type: *F*_(2,17070)_ = 346.1, *p*<0.0001; Time: *F*_(29,17070)_ = 0.11, *p* = 1.00) reduced cue-induced synchrony between DA-DA and DA-GABA pairs. Analyses include all neurons shown in **Figures 11**–**12**. Interestingly, while GABA-GABA synchrony showed no change relative to the reward cue, overall synchrony was elevated following pre-feeding treatment. These results suggest that cue-evoked synchrony, in addition to the magnitude of the phasic response to the reward cue, depends on an animal's motivational state.

## Discussion

We recorded from DA and GABA neurons in the substantia nigra as mice became progressively sated over the course of a 2-hr session, using a FT60s reinforcement schedule in which a food pellet was delivered once a minute. We found that phasic activity of both DA and GABA neurons in response to the reward cue (sound of the pellet dispenser) was bidirectionally modulated by motivational state (**Fig. 6**). For both cell types, we identified two distinct populations of neurons based on how motivational state changes their activity: those that increased their phasic response as the animal became sated, or “increasing neurons”, and those that decreased their phasic response, or “decreasing neurons".

The phasic response to the reward cue was largely eliminated when we prevented any changes in motivational state using extinction (**Figs. 10**–**12**). Extinction prevents satiety, but the lack of reward delivery may result in rapid learning that eliminated the cue-evoked phasic activity as the cue becomes an invalid predictor of reward. Therefore, it is difficult to determine from the extinction tests alone whether the observed modulation is caused by satiety. It is necessary to prevent a change in motivational state without changing the reward contingency. We therefore examined the effects of pre-feeding before the recording session, which should also prevent significant changes in motivational state by rapidly sating the mice. If the animal was already sated before the session, then there should not be significant changes in motivational state within the 2-hr session. We found that pre-feeding reduced the modulation of the cue-evoked response in decreasing DA and GABA neurons, and it also abolished any modulation of the phasic response of increasing neurons (**Figs. 11**–**12**). Together the data from the extinction and pre-feeding experiments suggest that the changes in DA and GABA activity in the SN may reflect changes in motivational state.

Even though extinction and pre-feeding tests are intended to prevent changes in motivational state [35], they also affect the behavior of the animals. Therefore, we cannot rule out the possibility that the observed modulation during FT60 sessions, the reduced modulation following pre-feeding, and the abolished phasic response during extinction are dependent on the behavior of the mice. Extinction and pre-feeding dramatically changed the mouse's behavior **(** **Fig. 8**). The reduced food-seeking behavior coincides with the reduction of the phasic response of both DA and GABA neurons to the reward cue. Since the rate of food cup entries was reduced by both pre-feeding and extinction, it is likely that the related changes in the phasic DA and GABA signals are indeed related to some component of behavior. One possibility is that following pre-feeding or during extinction, mice lost interest in the food pellets, no longer attending to the pellet-predicting cue. The specific relationship between behavior (i.e. locomotion, response vigor, attending to the cue) and neural output remains to be explored.

Interestingly, although the phasic response was abolished by extinction, it was not completely eliminated by pre-feeding. In contrast to the phasic response of decreasing neurons, the phasic response of increasing neurons was much more sensitive to pre-feeding (**Figs. 11b**, **12b**). While the phasic response of decreasing DA and GABA neurons was still present following pre-feeding, the phasic response of increasing neurons was completely eliminated. The functional implications of this disparity are unclear, but one possibility is that the output of these two populations of neurons (increasing and decreasing) reflects distinct contributions to behavior. This possibility remains to be tested.

Although the phasic response to the reward cue in DA neurons is similar to what has been reported previously [36], the observation of very similar phasic activity in GABA neurons in the SNR is novel. Indeed, the spiking of GABA neurons is often synchronized with that of DA neurons following the reward-predicting cue, which can be seen as cue-elicited synchronization among DA neurons and GABA neurons (**Fig. 5**). Thus DA neurons are not the only neurons that show this particular type of cue-elicited phasic activity, and whatever signal this phasic response represents cannot only be conveyed by DA, since GABA neurons in the SNR have a different pattern of anatomical connectivity. As these are putative projection neurons, the observed phasic activity should directly affect downstream structures in the thalamus and brainstem.

It is possible that the synchrony between DA and GABA neurons is driven by common inputs, e.g. from the striatum [3], [21], [29], [37]. It is also possible that synchrony is a result of electrical coupling. DA neurons, for example, are known to express gap junctions [38]. In addition, the dendritic release of DA can also depolarize GABA neurons by activating transient receptor potential (TRP) channels (in particular TRPC) [5], which is another potential mechanism for the observed effects. How satiety signals (hormonal or neural) can influence synaptic transmission and electrical coupling in the substantia nigra remains poorly understood, but our results suggest that they have a potent effect on the output of the basal ganglia as well as dopaminergic signaling.

In addition to the surprising similarity in the event-related firing patterns of DA and GABA neurons, we also observed some significant differences between these cell types. GABA neurons have much higher tonic firing rates than DA neurons, though their phasic response is more similar in magnitude. The baseline firing rate of GABA neurons was also more strongly modulated by motivational state (**Fig. 12a**), possibly because the tonic basal ganglia output more directly reflects the overall activity level of the animal.

Our results confirm and extend previous reports of reward-elicited synchrony in firing among midbrain dopamine neurons [31]. The observed synchrony cannot simply be explained by rate modulation: DA and GABA neurons show similar rate modulations following the reward cue, but DA-DA pairs exhibit significant changes in cue-elicited correlations whereas GABA-GABA pairs fail to show enhanced post-cue synchrony. Pre-feeding did not increase the firing rates of GABA neurons (**Fig. 12b**), but synchrony between GABA-GABA pairs was enhanced (**Fig. 14b**). The observed synchrony can therefore be independent of changes in firing rate.

Our finding that there are two distinct populations of DA neurons is in agreement with recent work [18], [36], [39]. Hikosaka and colleagues, for example, have shown that the activity of SNC DA neurons can be divided into two major classes: some neurons respond to predictors of reward but others respond to predictors of aversive outcomes [18]. An intriguing question is whether the DA neurons that are responsive to aversive outcomes are the same as those that increase phasic firing as animals become sated.

A prominent hypothesis is that phasic DA activity encodes a reward prediction error (RPE), based on electrophysiological studies in monkeys [36]. According to this hypothesis, phasic activity of DA neurons reflects the reward prediction error and serves as a teaching signal that regulates plasticity in regions receiving dopamine, e.g. striatum [36], [40], [41]. Although we replicated previous finding of phasic DA activity in response to the reward cue, our results cannot be explained by the RPE hypothesis.

The phasic DA activity we recorded does not reflect a reward prediction error. In our experiments, the prediction error is always the same because the reward cue always predicts the reward (except for the extinction sessions). Yet, DA activity changes systematically according to shifts in motivational state, with some increasing while others decreasing their response. In other words, there was no significant change in the predictability of the reward, only changes in motivational state.

One possible defense of the RPE hypothesis is that the “effective reinforcement” signal does not necessarily remain the same even if the physical reward remains the same, since the food can become less rewarding and presumably less effective as a reinforcement signal [42]. But even a modification of the RPE hypothesis to incorporate reduced subjective reward value cannot explain the present findings. The motivational modulation we observed is bidirectional: with satiety, some DA neurons increase their phasic response while others decrease their phasic response. This observation is incompatible with any version of the RPE hypothesis.

Interestingly, we found that DA neurons are not the only neurons showing cue-evoked phasic activity, as GABA neurons show a similar profile in relation to the reward predicting cue. Our recent work also found a similar pattern with an operant temporal differentiation task [22]. These GABA neurons are a major source of outputs from the basal ganglia, targeting many brain regions. The phasic response of these neurons is expected to directly contribute to the behavioral output, though in the present study we did not measure the behavioral output during the cue presentation. It would be crucial in future experiments to examine the contribution of such a signal–both from GABA and DA neurons–to behavior, though better and more precise behavioral measures will be required. Previous work on the role of DA has not carefully examined the behavior of the animal, often using very limited measures under highly artificial conditions. Further progress will depend on establishing the link between reward-related behavior, which is clearly modulated by motivational state [43], and the signals generated by the DA neurons and their target regions.

In accord with a growing body of work [33], [44], [45], DA neurons appear to be more important for the regulation of performance than for learning driven by prediction errors. The activity of both DA and GABA neurons reflects the motivational state of the animal and reward-seeking behavior. Given the widespread targets of SNC DA neurons and SNR GABA neurons, similar motivational modulation should also be found in the diverse brain regions receiving their projections, such as the cortex, striatum, brainstem, and thalamus. The obvious question is how nigral DA and GABA neurons receive information on the motivational state of the animal. Based on known anatomical connectivity, these signals can come from brainstem parabrachial nucleus and the hypothalamus [2], [46]. Hormones implicated in regulating the homeostatic system can also directly affect dopamine neurons; e.g., leptin and insulin can inhibit dopamine neurons, whereas ghrelin can excite them [4]. More detailed analysis of the anatomical projections and synaptic transmission between these regions is needed to elucidate these functional interactions. While many studies of reward processing have chosen to focus on the dopamine neurons located in the ventral tegmental area (VTA), relatively few have addressed the role of dopamine neurons of the substantia nigra in reward processing [22], [47]. Metabolic signals are known to interact with dopamine circuits in both the VTA and SN [4], [48]. As such it is possible that similar motivational modulation of neural activity could be observed in the VTA as well.
